# Longitudinal Evaluation of Neurological and Sensory Changes in Gaucher Disease: A Prospective Observational Cohort Study (SENOPRO)

**DOI:** 10.3390/medsci14020181

**Published:** 2026-04-02

**Authors:** Emanuele Cerulli Irelli, Adolfo Mazzeo, Nicoletta Fallarino, Francesca Caramia, Gianmarco Tessari, Enza Morgillo, Carlo Di Bonaventura, Rosaria Turchetta, Giovanna Palumbo, Maria Giulia Tullo, Laura Mariani, Marcella Nebbioso, Patrizia Mancini, Cecilia Guariglia, Fiorina Giona

**Affiliations:** 1 Department of Human Neurosciences, Sapienza University of Rome, 00185 Rome, Italy; emanuele.cerulliirelli@uniroma1.it (E.C.I.); adolfo.mazzeo@uniroma1.it (A.M.);; 2 Department of Psychology, Sapienza University of Rome, 00185 Rome, Italy; 3 Department of Translational and Precision Medicine, Sapienza University of Rome, 00185 Rome, Italy; 4 Department of Sense Organs, Sapienza University of Rome, 00185 Rome, Italy; 5 Department of Neuroscience, Imaging and Clinical Sciences, University G. d’Annunzio of Chieti-Pescara, Via dei Vestini 31, 66100 Chieti, Italy

**Keywords:** Parkinson’s disease (PD), dementia, hearing loss, memory, cognitive, Gaucher disease

## Abstract

**Background:** Gaucher disease (GD) is a rare lysosomal storage disorder caused by mutations in the GBA1 gene. Traditionally, GD is classified into three subtypes based on the severity of neurological involvement; however, overlapping clinical features increasingly suggest a continuum of phenotypes rather than distinct categories. In this prospective observational cohort study, we conducted a multidisciplinary assessment of patients with GD to identify and monitor neurological, cognitive, auditory, and visual impairments. **Materials and Methods**: A comprehensive clinical and instrumental evaluation was performed at baseline and repeated at follow-up, with a median interval of 37 months (IQR 36–38). Neurological assessments included physical examination, clinical rating scales, video-EEG, and brain MRI. Cognitive status was assessed using a standardized battery of neuropsychological tests. Detailed audiological and ophthalmological evaluations were also conducted. Paired parametric or non-parametric tests were applied as appropriate, with Bonferroni correction for cognitive outcomes (*p* < 0.05). **Results**: Of the 22 patients assessed at baseline, 18 completed the follow-up evaluation. Neurological assessments showed a worsening of subtle parkinsonian signs, with significant increases in Movement Disorder Society–Unified Parkinson’s Disease Rating Scale Part III scores (*p* = 0.04) and non-motor symptom scores (*p* = 0.01). Two of the eighteen patients developed epilepsy during follow-up. A high prevalence of sleep disturbances was confirmed, with 27.8% exhibiting excessive daytime sleepiness and 16.7% reporting REM sleep behaviour disorder on standardized questionnaires. Compared with baseline, cognitive assessments revealed a higher proportion of patients with performance below normative population scores in at least one cognitive domain, particularly memory. Sensorineural hearing loss was confirmed in 11 of 15 patients (73.3%) who underwent audiological evaluation, with progressive worsening of audiometric thresholds observed in 7 of 11 (64%). Ophthalmological evaluations showed no changes in visual acuity or OCT findings; however, multifocal electroretinography abnormalities were detected in 12 of 13 patients. **Conclusions**: Through in-depth phenotyping, this study identifies measurable neurological, cognitive, and sensory progressive changes in patients with GD over time, supporting the value of tailored, multidisciplinary long-term care strategies to monitor and address emerging clinical needs in this rare disease.

## 1. Background

Gaucher disease (GD) is a rare lysosomal storage disease, due to glucocerebrosidase (also known as glucosylceramidase or acid β-glucosidase; GBA) deficiency, caused by pathogenic variants in the GBA1 gene on the 1q21 locus. The insufficient enzymatic activity of GBA leads to the accumulation of its main substrate, glucosylceramide, along with other glycolipids, in the lysosomes of liver, spleen, and bone marrow reticuloendothelial cells [1].

The clinical presentation of GD is highly variable, and it can be classified into three subtypes according to the absence (type 1) or presence (type 2 and type 3) of neurological involvement. GD type 1 (GD1), classically defined as the ‘non-neuronopathic’ form, is the most common subtype, accounting for up to 95% of cases in the Western world, with a higher prevalence in individuals of Ashkenazi Jewish descent.

GD type 2 (GD2) is characterized by the most severe neurological phenotype, typically manifesting within the first months of life. It has a rapidly progressive course, leading to death in early childhood, and it is hence defined as ‘acute neuronopathic GD’. GD type 3 (GD3), conversely, represents the ‘chronic or subacute neuronopathic’ form and can be characterized by supranuclear gaze palsy, slow horizontal saccades, heterogeneous cognitive impairment (ranging from mild intellectual disability to progressive dementia), as well as epilepsy and myoclonus [2].

However, the presence of intermediate phenotypes and clinical profiles with overlapping features supports the view of GD as a continuum of disease manifestations rather than a condition limited to three clearly distinct subtypes [3,4,5]. Indeed, neurological symptoms are increasingly recognized as part of GD1, either in mild or subclinical forms, or as more severe neurological involvement, such as parkinsonism [6,7].

The involvement of multiple organs, combined with the variable severity of each manifestation, renders GD an extremely complex syndrome. It is challenging not only to diagnose but also to monitor, as diverse issues may arise at different stages throughout the disease course. Multidisciplinary care is essential, requiring specialized evaluations and numerous instrumental assessments to accurately identify early organ involvement and implement targeted treatments, thereby preventing further progression [8,9].

This challenge was highlighted in SENOPRO [10], a multidisciplinary investigation conducted in 2020, which included a detailed evaluation of 22 GD patients, 19 GD1 and 3 GD3, according to the traditional GD classification [5]. In this cohort, extensive neurological characterization revealed a significant prevalence of neurological symptoms, ranging from sleep disturbances to lower cognitive performances. Additionally, ad hoc assessments identified alterations in the auditory and visual systems among GD patients.

The primary aim of the current study was to investigate the longitudinal evolution of neurological, neuropsychological, and sensory alterations in GD patients at a three-year follow-up period. By employing a multidisciplinary approach, this study sought to provide deeper insights into the trajectory of these manifestations in GD.

## 2. Materials and Methods

This prospective observational cohort study, SENOPRO, enrolled patients with a genetically confirmed diagnosis of GD aged over 12 years, who were followed at AOU Policlinico Umberto I.

The original study design included three evaluations over a two-year period (baseline, 12 months, and 36 months). However, due to the COVID-19 lockdown, the 12-month follow-up evaluations could not be conducted. Instead, a multidisciplinary assessment was offered to all participants at approximately 36 months from baseline. The evaluation included hematological, neurological, neuropsychological, audiological, and ophthalmological assessments, consistent with those conducted at baseline [10].

The study was performed in compliance with the Declaration of Helsinki and was approved by the local ethics committee (protocol code: 2015/19; approval date: 21 February 2019). Informed consent was obtained from all participants at the start of the study.

### 2.1. Neurological Evaluation

Patients were interviewed by a trained neurologist, to assess the presence of sleep disturbances, such as restless legs syndrome and REM behaviour disorder (RBD), as well as the presence of other neurological symptoms (e.g., seizures). A thorough physical examination was also performed. Specifically, the presence of parkinsonian motor signs (bradykinesia, rigidity, and tremor) was noted, and the Movement Disorder Society-Unified Parkinson’s Disease Rating Scale (MDS-UPRDS) part III [11] was administered to all patients.

Standardized clinical scales to evaluate sleep disturbances and non-motor signs were also administered. The Epworth Sleepiness Scale was utilized to evaluate excessive daytime sleepiness (EDS), with a score above 10 indicating EDS [12]. Also, a RBD screening questionnaire (RBDSQ) was administered, with a score above 5 indicating a probable RBD [13,14].

To evaluate non-motor symptoms (NMS) associated with parkinsonism, the NMS score (NMSS) was employed. The NMSS examines 30 different NMS, organized into 9 distinct domains: cardiovascular autonomic instability, sleepiness/fatigue, mood/apathy, perceptual issues, memory/attention, gastrointestinal disturbances, urinary tract dysfunction, sexual activity, and miscellaneous symptoms (such as pain unrelated to known medical conditions, hyposmia, sweating disturbances, and unexplained weight changes).

Video-electroencephalogram (video-EEG) was repeated in all patients, using a Micromed System Plus 21-channel device (Mogliano Veneto, Italy), according to the 10–20 International System. Finally, for patients who consented to repeat neuroimaging, MRI was performed on a 3T Siemens MAGNETOM Verio scanner (Erlangen, Germany) equipped with a twelve-channel phase-array head coil.

### 2.2. Neuropsychological Assessment

The Mini-Mental State Examination (MMSE) [15] was administered to assess global cognitive function. Additionally, patients underwent a comprehensive neuropsychological battery, including Rey’s Auditory Verbal Learning Test [16], Immediate Visual Memory Test [16], Corsi Block-Tapping Test [17], Digit Span Test [17], and Babcock Story Recall for the memory domain [18]. For the attentional domain, the battery included the Multiple Features Target Cancellation Test (Toulouse-Piéron) [19] and the Trail-Making Test [20]. Language and executive functions were evaluated using Word Fluency [16] and Phrase Construction tasks [16]. Visuo-constructional abilities were assessed through Freehand Copying of Drawings and Copying Drawings with Landmarks [16], while abstract reasoning was measured with Raven’s Progressive Colored Matrices [16]. Furthermore, prior to the neuropsychological assessment, each participant was interviewed and evaluated using the Brief Psychiatric Rating Scale [21].

### 2.3. Hearing Evaluation

A full audiological evaluation was conducted. Pure tone audiometry was administered at octave frequencies from 125 to 8000 Hz using frequency-modulated tones in a soundproof booth, employing an Auricle audiometer (Otometrics, Copenhagen, Denmark) with TDH39 headphones. Speech perception in quiet was measured using balanced sentence lists from the Italian Speech Audiometry [22], presented at 65 dB SPL from a frontal speaker, with scores ranging from 0 to 100%. Speech perception in noise was assessed using the Italian-adapted Matrix Test [23]. Finally, Auditory Brainstem Response (ABR) testing was performed, with recordings conducted in a soundproof room using an Interacoustics Eclipse EP25 (Interacoustics A/S, Middelfart, Denmark) at a click rate of 21 stimuli per second and an analysis window of 12 ms.

### 2.4. Ophthalmological Evaluation

The ophthalmological assessment included an orthoptic examination, a slit-lamp evaluation, and the measurement of best-corrected visual acuity using the Early Treatment Diabetic Retinopathy Study charts at a distance of four meters. Additionally, intraocular pressure was measured, and Visual evoked potentials (VEPs) and full-field electroretinography (ERG) were conducted. Optical coherence tomography (OCT) was used to evaluate retinal structure in detail, and multifocal ERG (mfERG) was performed to assess localized retinal function across the central visual field.

### 2.5. Statistical Analysis

The normality of the data was evaluated using visualization techniques and the Shapiro–Wilk test. Depending on the data distribution, results were summarized as mean values for normally distributed data or as medians for non-normally distributed data. Statistical comparisons within groups (involving the same subjects at different time points) were carried out using the paired t-test for normally distributed data and the Wilcoxon Signed-Rank Test for non-normally distributed data. For the follow-up cognitive assessment, given the high number of tests performed, results were adjusted for multiple comparisons, and Bonferroni-adjusted *p*-values are presented. All statistical analyses were conducted using SPSS software, version 27, with a *p*-value of less than 0.05 considered statistically significant. Plots were generated using the ggplot package in R version 4.2.3.

## 3. Results

### 3.1. General Clinical Features

Out of the twenty-two patients enrolled in the first evaluation, two died due to their underlying neurological conditions. Specifically, one patient with GD1 died from complications related to advanced Parkinson’s Disease (PD), and one patient with GD3 died from complications of motor neuron disease. Additionally, two other patients (one with GD1 and one with GD3) refused to participate in the follow-up study. A complete flow chart of the study participants is shown in the Supplementary Material (Figure S1).

As a result, eighteen patients (seventeen GD1 and one GD3; seven male and eleven female) proceeded with the follow-up evaluation conducted between January 2022 and May 2023. The median interval between the first and second evaluation was 37 months (Interquartile range, IQR 36–38 months), and the median age at the time of evaluation was 47 years (IQR 38–60). All patients but one were undergoing treatment, with a median treatment duration of 21 years (IQR 9.4–24.5).

Regarding GBA1 genotype, the N370S [also known as p.(N409S)] variant was present in 16 individuals (88.9%), most frequently in combination with L444P [also known as p.(L483P)] (6 cases). Other compound heterozygous combinations included N370S with RecNcil (2 cases), P99Lfs*62 (2 cases), c.1389-1G > A (1 case), 2123insA (1 case), R285H (1 case), W184R (1 case), G202R (1 case), and a complex mutation (1 case [24]). Additionally, one participant exhibited the L444P/L444P genotype (the only patient with GD3), and another carried the complex L444P + V460V/RecFs variant.

The genotype of each patient and the results of the multidisciplinary assessment are reported in Table 1, using patient codes consistent with those from the baseline study [10].

### 3.2. Neurological Findings

Neurological assessment was repeated in 18 of the 22 patients who had been evaluated at baseline.

Among the 18 patients who completed the follow-up assessment, no new diagnosis of parkinsonism was made. However, subtle parkinsonian signs (such as appendicular bradykinesia, often minimal, without rigidity or tremor) were noted in a higher percentage of patients (27% vs. 55.5%), with a significant increase in the median MDS-UPRDS part III score from baseline to the follow-up at 3 years (0 [IQR 0–5] vs. 3 [IQR 1.5–6], *p* = 0.04).

**Figure 1:**
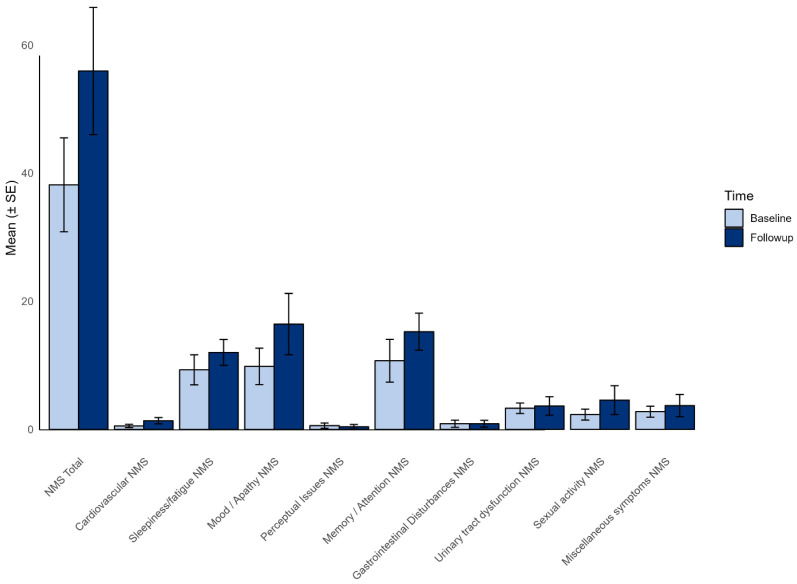
Changes in non-motor symptom scores from baseline to follow-up. The bar chart presents the mean scores (±SE) for various non-motor symptoms (NMS) sub-items at baseline and follow-up.

Likewise, a significant difference was noted regarding NMS, with a significant increase in the mean NMS rating score at 3 years (38.2 ± 31.1 vs. 51.7 ± 43.5, *p* = 0.01). Detailed results of the total NMSS along with scores of sub-items compared with baseline evaluation are reported in Figure 1 (complete values are provided in Supplementary Table S1).

In one patient who presented with severe parkinsonism and psychosis (pt#3), pharmacological therapy was adjusted by replacing olanzapine with clozapine. This change led to an improvement in motor function and cognitive performance without exacerbating the psychotic symptoms.

On follow-up electroencephalogram (EEG), interictal epileptiform discharges according to the International Federation of Clinical Neurophysiology criteria [25] were identified in four additional patients. In one case (pt#20, with GD3), a right temporal-onset seizure characterized by consciousness impairment and oral automatisms was recorded on EEG. This patient reported both focal seizures and focal to bilateral tonic–clonic seizures, leading to a diagnosis of focal epilepsy, with no evidence of structural lesions on brain MRI. Epilepsy (undetermined whether focal or generalized) was also diagnosed in a GD1 patient (pt#17) who presented with a single unprovoked morpheic seizure after some days of partial sleep deprivation, together with rare epileptiform discharges intermingled with physiological sleep figures on sleep EEG. Anti-seizure medication was initiated, achieving seizure freedom in the GD1 patient with levetiracetam at 1000 mg/day. In contrast, the GD3 patient was treated with lacosamide at 400 mg/day, with a suboptimal response. Indeed, rare focal seizures persisted during sleep, but the patient decided not to initiate add-on therapy.

Regarding sleep disturbances, a high prevalence of EDS (5/18, 27.8%) and restless legs syndrome (7/18, 38.8%) was confirmed during the three years of follow-up, although a slight decrease was observed for the prevalence of EDS. However, two more patients were diagnosed with probable RBD based on the RBDSQ, increasing the total to three (3/18, 16.7%). One patient (pt#19), who had been diagnosed with idiopathic central hypersomnia, was treated with solriamfetol and vortioxetine, experiencing a significant improvement in EDS and in quality of life. One patient with severe restless legs syndrome was treated with gabapentin with partial benefit.

No new findings were observed on neuroimaging in those patients who agreed to repeat a brain MRI (13/18 patients).

### 3.3. Neuropsychological Findings

A comprehensive neuropsychological evaluation was performed at follow-up in 17 of the 22 patients assessed at baseline, 1 with GD3 and the remainder with GD1. At the second evaluation, no new cases of dementia or mild cognitive impairment were identified. The mean MMSE values were comparable between the two time-points (27.7 ± 4.7 vs. 27.4 ± 3.9, *p* = 0.824).

However, in-depth cognitive assessments at follow-up revealed that 8 out of the 17 patients evaluated (47.1%) scored below the 5th percentile in at least one cognitive domain. In contrast, three years earlier, the prevalence of patients with significantly low performance in at least one cognitive domain was slightly lower (31.8%). The most frequently affected cognitive domain was verbal memory (7 out of 8, 87.5%).

**Figure 2:**
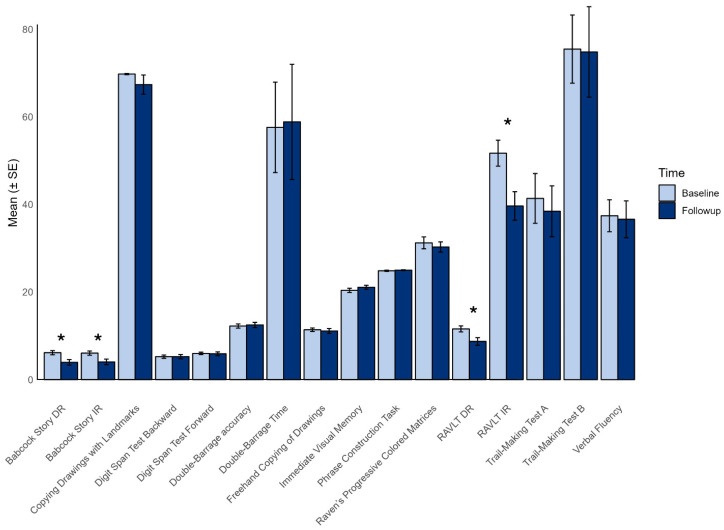
Comparison of raw cognitive scores at baseline and follow-up. The bar chart presents the mean scores (±SE) for various cognitive tasks at baseline and follow-up. Asterisks indicate significant differences after Bonferroni adjustment. Abbreviations: DR = delayed recall; IR = immediate recall. RAVLT = Rey Auditory Verbal Learning Test.

Indeed, from baseline to follow-up evaluation, there was a significant worsening in the immediate and delayed recall of Rey’s 15 words test (raw score: 51.7 ± 11.5 vs. 39.7 ± 12.6, *p* = 0.002; and 11.6 ± 0.67 vs. 8.7 ± 0.87, *p* = 0.006) and in both the immediate and delayed recall of the Babcock Story (raw scores: 6.1 ± 1.9 vs. 4.05 ± 2.7, *p* = 0.006; and 6.2 ± 1.9 vs. 3.95 ± 2.6, *p* = 0.002), as shown in Figure 2. No significant differences in other domains were observed. The raw scores per each test are shown in Supplementary Table S2.

Regarding psychiatric comorbidities, the prevalence of depression and somatic concerns remained stable. However, anxiety was diagnosed in two more patients, increasing the total to 7 out of 18 (38.9%). No association was found between psychiatric comorbidities and the presence of cognitive impairment.

### 3.4. Hearing Findings

Follow-up audiological assessment was performed in 15 of the 22 patients who had been evaluated at baseline. While no new subjects developed hearing loss, sensorineural hearing loss (SNHL) was confirmed in all patients in whom it had been identified at baseline (11/15 patients, 73.3%, 10 patients with GD1 and 1 patient with GD3).

The audiometric profile was stable in 4 of the 11 patients (36%) while 5/11 (46%) exhibited a slight worsening in the audiometric profile and 2/11 (18%) exhibited a severe worsening. Of the two patients with severe worsening, one (pt#15) progressed from mild SNHL to severe SNHL in one ear and anacusis in the other. The second (pt#14) transitioned from bilateral mild SNHL to moderate SNHL in one ear and mild SNHL in the other.

Audiogram patterns remained identical in all individuals: 6/11 patients showed a down-sloping audiogram; 3/11 patients presented a cookie-bite or a reverse cookie-bite audiogram, while the remaining 2 patients presented a flat audiogram. Regarding the severity of hearing loss, on average, patients had mild down-sloping hearing loss as measured by pure tone audiometry across different frequency ranges: 27.7 dB (±28.4) in the right ear and 24.3 dB (±17.6) in the left ear for 125–250 Hz; 27.4 dB (±27.4) in the right ear and 25.8 dB (±18.7) in the left ear for 500–2000 Hz; 32.9 dB (±32.5) in the right ear and 35.4 dB (±21) in the left ear for 4000–8000 Hz.

All three patients with the cookie-bite audiogram presented a slight worsening of the audiometric profile, with the average overall thresholds across all frequencies (125–8000 Hz) increasing from 15.8 dB (SD 4.3) to 23.6 dB (SD 3.8) in the right ear, and from 15.5 dB (SD 4.6) to 25.2 dB (SD 2.9) in the left ear.

The average speech perception score for sentences in quiet was 95.3% (±18), while in noise, it was 91.4% (±25.5). The average Matrix score was −4.8 dB SNR (±7). All patients, except for one with severe hearing loss, achieved 100% speech perception comprehension in quiet. However, four patients with mild-to-severe hearing loss showed a reduction in speech comprehension in noise. The Matrix test showed a slight improvement compared to the initial evaluation, likely attributable to test training.

Finally, the mean ABR wave thresholds were 23.5 dB HL (±7.4) for the right ear and 31.3 dB HL (±14) for the left ear. A positive correlation was observed between pure-tone average at the 4000–8000 Hz threshold and ABR wave V latency (rho = 0.72, *p* < 0.001 for the right ear; rho = 0.90, *p* < 0.001 for the left ear).

### 3.5. Ophthalmological Findings

Of the 22 patients assessed at baseline, 18 underwent follow-up ophthalmological evaluations. Comparison between the two time points revealed no clinically qualitative changes in visual acuity, corneal status, fundus and OCT findings. However, alterations in VEP, specifically a reduction in amplitude of the P100, were noted in two additional patients, reaching a total of 14 patients (77.8%).

Moreover, 13 patients also underwent mfERG in both eyes. All patients but one presented at least one eye showing an altered response on mfERG. Specifically, five eyes had one altered ring, seven eyes had two altered rings, one eye had three altered rings, and seven eyes had all the rings altered.

## 4. Discussion

In this study, we conducted a comprehensive, multidisciplinary follow-up assessment of patients with GD initially evaluated three years earlier [10]. Our findings revealed measurable changes over time across multiple domains, including neurological, cognitive, and sensory functions, further supporting the concept of GD as a disease continuum, irrespective of its subtype.

In our cohort, we observed a significant increase in the prevalence of subtle parkinsonian motor symptoms, contributing to the growing body of evidence linking GBA1 mutations to alpha-synuclein neurodegenerative pathways [7,26,27]. Both heterozygous GBA carriers and patients with GD are considered genetically at risk for PD, with more severe mutations associated with a higher risk [28]. Although none of the patients in this study phenoconverted to a definite PD diagnosis, we observed a significant increase in the burden of subtle parkinsonian motor signs and non-motor symptoms over the 3-year follow-up. Furthermore, we also observed an increased prevalence of probable RBD—a well-established prodromal manifestation of PD [29].

Our results align with previous longitudinal studies of both heterozygous GBA1 carriers and GD patients, which documented significant worsening of both motor and non-motor symptoms over time, with a higher burden in GD patients compared to heterozygous carriers [30,31]. However, all these studies—including the 6-year follow-up by Avenali et al. [27], in which only one of 31 GD patients developed PD—have consistently reported a low rate of phenoconversion, underscoring the need for further investigation into the clinical relevance of this subclinical progression.

At baseline, none of the GD patients had epilepsy. However, recurrent unprovoked seizures, accompanied by epileptiform abnormalities, led to an epilepsy diagnosis in two individuals (one GD1 and one GD3) during follow-up. The literature on epilepsy is currently limited to GD3 patients, ranging from benign forms, characterized by absence seizures or generalized tonic–clonic seizures, to more severe phenotypes, such as progressive myoclonic epilepsy [8,32,33,34,35,36,37,38]. Interestingly, in our cohort, one patient with GD1 developed epilepsy, albeit with a later onset compared to the patient with GD3 (45 years vs. 28 years). While a coincidental association between GD1 and epilepsy in our patient cannot be ruled out, we might speculate that individuals with GD1 may have an increased risk of developing late-onset epilepsy. This possibility is further supported by a recent case report describing a 75-year-old patient with GD1 who developed myoclonic seizures [34]. Consistent with this hypothesis, in our cohort, we also observed a higher prevalence of epileptiform abnormalities on follow-up EEGs.

Regarding the neuropsychological profile, to our knowledge, this is the first longitudinal study assessing cognitive functions in GD through in-depth cognitive testing. We documented worsening performances in the memory domain, with significantly lower verbal memory scores across time. In our previous study [10], memory impairment was significantly associated with reduced hippocampal thickness. Similarly, prior studies had found memory and attention to be the most commonly affected domains in GD [30,39]. Interestingly, in our cohort, the memory domain of the NMS scale was also amongst the most affected, suggesting an increased burden of both subjective and objective impairment in our cohort.

Notably, according to the literature, patients with GBA1-related PD (GBA1-PD) are characterized by a higher prevalence of dementia compared to those with non-GBA1-PD [40,41,42,43], suggesting a role of GBA1-mediated pathways in the genesis of cognitive impairment.

Additionally, the presence of SNHL was identified in up to 73% of our GD patients, with severity ranging from mild to severe. In the existing literature, hearing loss has not been extensively described in GD patients, with prior sparse reports primarily involving GD2 and GD3 phenotypes [44,45]. Our follow-up study confirms the presence of SNHL also in patients with GD1 and highlights that, in some patients, hearing loss may progress, potentially reducing quality of life. While the underlying mechanism of hearing involvement remains unknown, our findings highlight the importance of in-depth audiological evaluation in GD management.

Furthermore, considering that hearing loss is a risk factor for dementia and mild cognitive impairment [46], as well as for PD according to a recent report [47], future studies should investigate whether the increased prevalence of SNHL is confirmed in larger cohorts of patients with GD and whether its management may mitigate the progression of motor and cognitive symptoms.

Our comprehensive ophthalmic and neurophysiological assessments confirmed the presence of subclinical visual system alterations in up to 78% of GD patients. While eye involvement in GD is heterogeneous, sight-threatening conditions remain rare [48]. A few studies have identified alterations in visual evoked potentials, suggesting that these may serve as early indicators of subclinical neurological involvement [49,50]. Our study is the first to evaluate the GD population with mfERG. Rare reports had previously suggested functional retinal changes in GD, possibly due to glucosylceramide accumulation in retinal glial cells [51]. We identified altered responses in mfERG in 12 of the 13 patients tested. These findings, along with the normal full-field ERG responses observed in our previous study [10], may suggest a subclinical alteration in the macular region of the retina of GD patients [52]. 

It is worth noting that our cohort exhibited a high prevalence of severe GBA1 variants, including missense mutations (e.g., L444P, R285H, W184R, D409H, and G202R) as well as complex recombinant alleles arising from gene–pseudogene rearrangements involving GBAP1 (e.g., RecNciI). This genotypic background may have contributed to the progression of neurological and sensory abnormalities observed in this study, as suggested by previous research linking severe mutations to greater neurological impairment [28,53,54].

The main strengths of this study lie in its comprehensive multidisciplinary assessment and prospective design. However, the small sample size as well as the heterogeneity of patients at baseline represent a significant limitation, potentially introducing confounding and limiting the interpretability of pooled analyses. Notably, two patients declined the follow-up evaluation, and a few patients did not complete all instrumental assessments, highlighting the challenges of conducting such a comprehensive assessment in routine clinical practice. Additionally, the lack of a matched control group prevented us from determining whether the observed progression of neurosensory alterations is attributable to GD solely, rather than to aging, to the presence of other genetic risk factors, or to the exposure to external unknown *noxae*. Furthermore, the absence of fluid biomarker assessments represents an additional limitation, as such measures could have provided complementary insights into disease mechanisms and may be valuable to incorporate in future longitudinal studies.

## 5. Conclusions

In conclusion, this multidisciplinary and longitudinal study suggests the possibility of progressive changes in the neurological, auditory, and visual systems of patients with GD. In our cohort, these changes ranged from significant impairments to subclinical findings detectable only through instrumental evaluations. Overall, although our findings are limited by baseline heterogeneity and the lack of healthy controls, they contribute to a deeper understanding of the longitudinal trajectory of GD.

These insights highlight the importance of a multidisciplinary approach to patient care and provide useful data for future randomized controlled trials aimed at evaluating the full range of GD manifestations.

## Figures and Tables

**Table 1:** Neurological and sensory abnormalities in each patient at follow-up evaluation.

ID	Age (Decade of Life) */Sex	GBA1 Variants	Last Therapy, Duration (y)	Neurological	Neuropsychological	Visual	Auditory
#1	30–40/M	N370S/ L444P	Imiglucerase, 25 y	Subtle parkinsonian signs, RLS	Normal	VEP: reduced amplitude and increased latency; mfERG: abnormal central 25° both eyes	Normal, stable
#2	50–60/M	N370S/ L444P	No treatment	Subtle parkinsonian signs	MCI: memory	VEP: reduced amplitude and increased latency; mfERG: n.a.	Slightly worsening SNHL
#3	40–50/F	N370S/ L444P	Imiglucerase, 26 y	Parkinsonism, RLS	Dementia, psychosis	Normal	n.a.
#4	F	N370S/ L444P	Died at 46 years of age due to complications of parkinsonism				
#5	F	N370S/L444P	Refused follow-up evaluation				
#6	50–60/F	N370S/ L444P	Velaglucerase alfa, unk	Subtle parkinsonian signs, EDS, RLS	MCI: memory	VEP: normal; mfERG: abnormal central 5° both eyes	Stable SNHL
#7	40–50/F	N370S/ L444P	Velaglucerase, 6 y	Subtle parkinsonian signs, RLS	MCI: memory; anxiety	VEP: reduced amplitude; mfERG: abnormal central 2° L eye and 5° R eye	Normal, stable
#8	40–50/M	N370S/ L444P	Imiglucerase, 8 y	Normal	MCI: memory; anxiety, depression	VEP: reduced amplitude; mfERG: abnormal central 5° L eye	Stable SNHL
#9	60–70/M	N370S/ RecNcil	Eliglustat, 3 y	Subtle parkinsonian signs	Multidomain MCI: memory, attention	VEP: reduced amplitude; mfERG: abnormal central 5° both eyes	Slight worsening SNHL
#10	50–60/F	N370S/ RecNcil	Imiglucerase, 13 y	EDS, probable RBD	Anxiety	VEP: reduced amplitude; mfERG: abnormal central 15° R eye, 2° L eye	Slight worsening SNHL: cookie and reverse cookie bite
#11	20–30/F	N370S/P99Lfs*62	Imiglucerase, 8 y	EDS	Anxiety, somatic concerns	VEP: normal; mfERG: n.a.	n.a.
#12	20–30/M	N370S/ P99Lfs*62	Imiglucerase, 4 y	Normal	Multidomain MCI: memory, executive functions	VEP: normal; mfERG: abnormal central 25° L eye	Normal, stable
#13	50–60/F	N370S/c.1389-1G > A	Eliglustat, 2 y	Subtle parkinsonian signs, probable RBD, RLS	MCI: attention; somatic concerns	VEP: reduced amplitude; mfERG: abnormal central 5° L eye	Stable SNHL
#14	60–70/F	N370S/ complex	Eliglustat, 4 y	RLS	Normal	VEP: reduced amplitude; mfERG: abnormal central 5° R eye, central 10° L eye	Severe worsening SNHL
#15	60–70/F	N370S/ 2123insA	Velaglucerase alfa, 13 y	Parkinsonism	Dementia	VEP: increased latency; mfERG: abnormal central 25° both eyes	Severe worsening SNHL
#16	70–80/F	N370S/ R285H	Imiglucerase, 23 y	Subtle parkinsonian signs	n.a.	VEP: normal; mfERG: n.a.	n.a.
#17	40–50/F	N370S/ W184R	Imiglucerase, 9 y	Epilepsy	Anxiety, somatic concerns	VEP: normal; mfERG: n.a.	Slight worsening SNHL: cookie and reverse cookie bite
#18	40–50/F	N370S/G202R	Velaglucerase alfa, unk	Probable RBD	MCI: memory; anxiety, depression	VEP: normal; mfERG: normal	Normal, stable
#19	30–40/M	L444P + V460V/RecFs	Eliglustat, 4 y	Subtle parkinsonian signs, central hypersomnia	Anxiety, somatic concerns	VEP: reduced amplitude; mfERG: abnormal central 25° both eyes	Slight worsening SNHL: cookie and reverse cookie bite
#20	20–30/M	L444P/L444P	Velaglucerase alfa, 14 y	Subtle parkinsonian signs, saccadic impairment, epilepsy, RLS	Multidomain MCI: memory, attention, language, executive functions	VEP: reduced amplitude; mfERG: abnormal central 2° both eyes	Stable SNHL of variable shape
#21	M	L444P/ L444P	Refused follow-up evaluation				
#22	M	D409H/D409H	Died at 35 years of age due to complications of motor neuron disease				

Abbreviations: mfERG = multifocal electroretinography; n.a. = not acquired; RBD = REM behavior disorder; RLS = restless legs syndrome; SNHL = sensorineural hearing loss; VEP = visual evoked potential. * To protect participant anonymity, age is reported by decade of life rather than as exact values.

## Data Availability

The data presented in this study are available on request from the corresponding author. The data are not publicly available due to privacy restrictions.

## Supplementary Materials

The following supporting information can be downloaded at: https://www.mdpi.com/article/10.3390/medsci14020181/s1, Figure S1: Flow diagram of patient inclusion and completion of follow-up evaluations; Table S1: Cognitive outcomes at baseline and follow-up; Table S2: Non-motor symptoms (NMS) at baseline and follow-up.

## Abbreviations

ABR	Auditory Brainstem Response
EDS	Excessive daytime sleepiness
EEG	Electroencephalogram
ERG	Electroretinography
GBA1-PD	GBA1-related Parkinson’s disease
GD	Gaucher Disease
GD1	Gaucher Disease type 1
GD2	Gaucher Disease type 2
GD3	Gaucher Disease type 3
HL	Hearing loss
IQR	Interquartile Range
MDS-UPRDS	Movement Disorder Society-Unified Parkinson’s Disease Rating Scale
mfERG	Multifocal electroretinography
MMSE	Mini-Mental State Examination
NMS	Non-motor symptoms
NMSS	Non-motor symptoms score
OCT	Optical coherence tomography
PD	Parkinson’s Disease
RBD	REM behaviour disorder
RBDSQ	REM behaviour disorder screening questionnaire
SNHL	Sensorineural hearing loss
VEPs	Visual evoked potentials
