# Supplementary material for: Longitudinal Evaluation of Neurological and Sensory Changes in Gaucher Disease: A Prospective Observational Cohort Study (SENOPRO)
Source: Med Sci (Basel). 2026 Apr 2;14(2):181. doi: 10.3390/medsci14020181 (PMC13108146; doi:10.3390/medsci14020181)

Supplementary Table S1. Cognitive outcomes at baseline and follow-up (mean ± SE).

Test	Baseline Mean ± SE	Follow-up Mean ± SE	Δ	% Change
RAVLT IR	51.73 ± 2.96	39.67 ± 3.26	-12.06	-23.3%
RAVLT DR	11.60 ± 0.69	8.73 ± 0.87	-2.87	-24.7%
Babcock Story IR	6.06 ± 0.48	4.05 ± 0.64	-2.01	-33.2%
Babcock Story DR	6.16 ± 0.47	3.95 ± 0.63	-2.21	-35.9%
Verbal Fluency	37.44 ± 3.64	36.63 ± 4.20	-0.81	-2.2%
Phrase Construction Task	24.87 ± 0.13	25.00 ± 0.10	+0.13	+0.5%
Raven's Progressive Matrices	31.25 ± 1.36	30.31 ± 1.17	-0.94	-3.0%
Immediate Visual Memory	20.40 ± 0.49	21.10 ± 0.45	+0.70	+3.4%
Freehand Copying	11.40 ± 0.41	11.13 ± 0.56	-0.27	-2.4%
Copying with Landmarks	69.80 ± 0.14	67.40 ± 2.19	-2.40	-3.4%
Trail-Making Test A	41.40 ± 5.69	38.47 ± 5.81	-2.93	-7.1%
Trail-Making Test B	75.50 ± 7.78	74.86 ± 10.34	-0.64	-0.8%
Double-Barrage Accuracy	12.25 ± 0.49	12.50 ± 0.60	+0.25	+2.0%
Double-Barrage Time	57.63 ± 10.32	58.88 ± 13.16	+1.25	+2.2%
Digit Span Forward	6.00 ± 0.28	5.92 ± 0.43	-0.08	-1.3%
Digit Span Backward	5.25 ± 0.37	5.25 ± 0.48	0.00	0.0%

Abbreviations: DR = delayed recall; IR = immediate recall. RAVLT = Rey Auditory Verbal Learning Test.

Supplementary Table S2. Non-motor symptoms (NMS) at baseline and follow-up (mean ± SE).

Domain	Baseline Mean ± SE	Follow-up Mean ± SE	Δ	% Change
NMS Total	38.17 ± 7.34	55.94 ± 9.94	+17.78	+46.6%
Cardiovascular NMS	0.53 ± 0.26	1.35 ± 0.49	+0.82	+154.7%
Sleepiness/Fatigue	9.29 ± 2.35	12.00 ± 2.02	+2.71	+29.2%
Mood/Apathy	9.82 ± 2.84	16.41 ± 4.77	+6.59	+67.1%
Perceptual Issues	0.59 ± 0.41	0.41 ± 0.35	-0.18	-30.5%
Memory/Attention	10.71 ± 3.33	15.24 ± 2.89	+4.53	+42.3%
Gastrointestinal	0.88 ± 0.56	0.88 ± 0.53	0.00	0.0%
Urinary dysfunction	3.29 ± 0.82	3.65 ± 1.44	+0.35	+10.7%
Sexual activity	2.31 ± 0.85	4.56 ± 2.26	+2.25	+97.4%
Miscellaneous symptoms	2.76 ± 0.86	3.71 ± 1.73	+0.95	+34.4%

Supplementary Figure S1. Flow diagram of patient inclusion and completion of follow-up evaluations.

Abbreviations: GD1 = Gaucher disease type 1; GD3 = Gaucher disease type 3; mfERG = multifocal electroretinography; MRI = Magnetic Resonance Imaging.

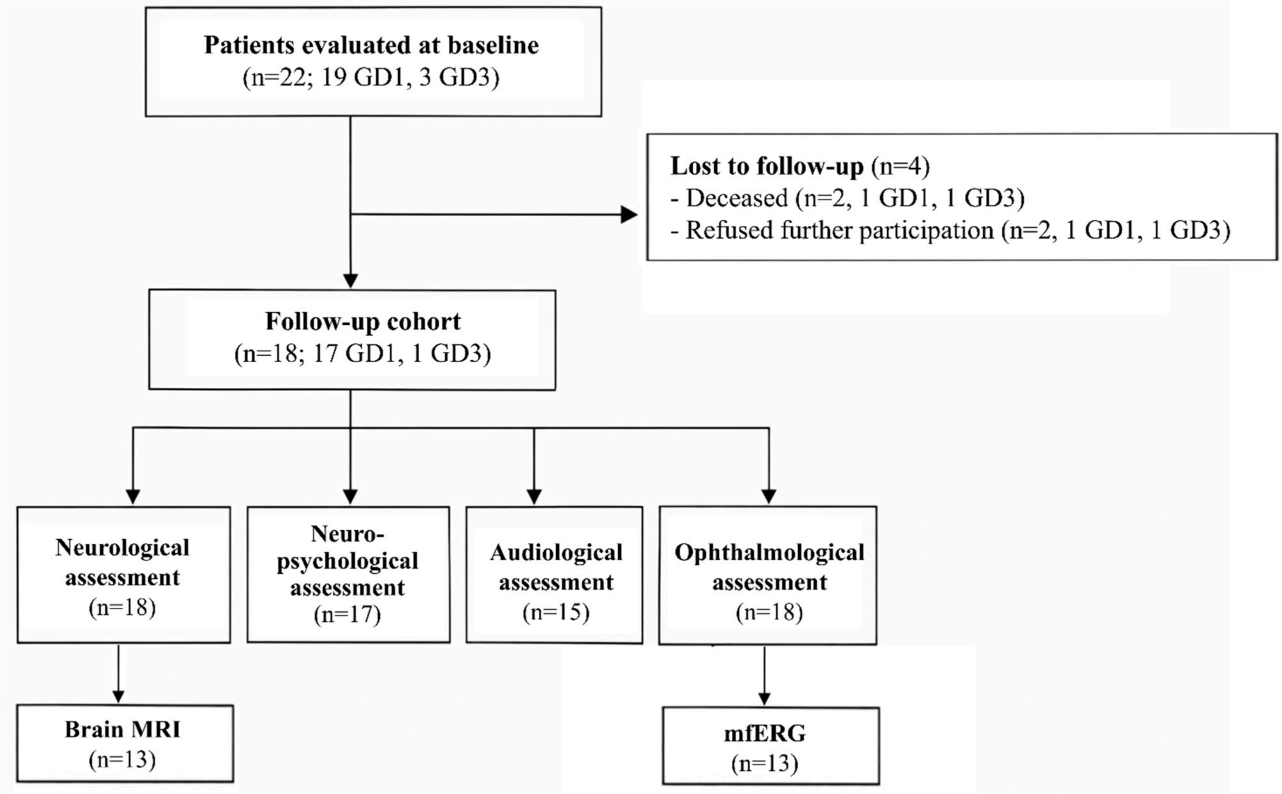

Supplement: Supplementary file 1 [file medsci-14-00181-s001.zip › medsci-4194260-supplementary.pdf]
